# Safety and tolerability of intravitreal cetuximab in young and adult rabbits

**DOI:** 10.1038/s41598-022-15642-4

**Published:** 2022-07-06

**Authors:** Mukharram M. Bikbov, Gyulli M. Kazakbaeva, Songhomitra Panda-Jonas, Dinar A. Khakimov, Leisan I. Gilemzianova, Liana A. Miniazeva, Azaliia M. Tuliakova, Albina A. Fakhretdinova, Renat A. Kazakbaev, Ildar F. Nuriev, Jost B. Jonas

**Affiliations:** 1 Ufa Eye Research Institute, 90 Pushkin Street, Ufa, Bashkortostan, Russia 450008; 2 Ural Eye Institute, Ufa, Russia; 3 Privatpraxis Prof Jonas Und Dr Panda-Jonas, Heidelberg, Germany; 4 Department of Ophthalmology, Medical Faculty Mannheim, Heidelberg University, Theodor-Kutzerufer 1, 68167 Mannheim, Germany; 5 Institute of Molecular and Clinical Ophthalmology, Basel, Switzerland

**Keywords:** Drug discovery, Drug safety, Drug development

## Abstract

To assess safety and tolerability of intraocularly applied cetuximab as epidermal growth factor receptor antibody, we conducted the experimental study which consisted of groups of adult rabbits (body weight: 2.4 kg) and young rabbits (body weight: 1.6 kg). All animals received three intravitreal injections of 0.5 mg cetuximab (Erbitux) (0.10 mL; 5 mg cetuximab/mL) into their right eyes in 4-week intervals, while the contralateral eyes received intravitreal injections of Ringer's solution. All animals underwent regular ophthalmological examinations at baseline and two-week intervals. Four weeks after the last injection, the animals were sacrificed, and the eyes were enucleated, fixed and examined by light microscopy. The study included 10 adult rabbits (age: 18 weeks; range: 17–19 weeks) and 8 young rabbits (age: 8 weeks; range: 7–10 weeks). Biometric measurements of axial length, anterior chamber depth and lens thickness and intraocular pressure readings did not differ significantly (all *P* > 0.05, Bonferroni corrected) between the right (study) eyes and the left (control) eyes, neither in the young nor in the adult rabbit group. Signs of intraocular inflammation or fundus peculiarities were not detected. Thickness of the outer nuclear layer, inner nuclear layer, combined outer and inner nuclear layer and outer plexiform layer, and total retina, measured at the posterior pole, posterior pole/equator midpoint, equator, and ora serrata region, did not vary significantly between study eyes and control eyes (all *P* > 0.05, Bonferroni corrected). The results suggest that repeated intravitreal application of cetuximab did not result in any detected intraocular toxic or destructive effect in young and adult rabbits, concurring with the notion of an intraocular tolerability of cetuximab.

## Introduction

The mechanism of ocular axial elongation during the process of emmetropization has remained elusive so far. Emmetropization describes the change from the newborn's marked axial hyperopia to emmetropia in the young adult. In the case of an overshooting of the process, axial myopia results. While in moderately myopic individuals, axial myopization usually stops in the third decennium, recent studies have revealed that in adult highly myopic patients, axial elongation can continue and constitute a major risk factor for the progression of myopic macular degeneration^1–4^. Histomorphometric, immunohistochemical, experimental and clinical studies have suggested that epidermal growth factor (EGF) may be involved in the process of axial elongation^5–8^. Young guinea pigs, with or without lens-induced myopization, showed an increased axial elongation when EGF or EGF family members were applied intravitreally^6–8^. As a corollary, intravitreal application of antibodies against EGF and EGF family members such as amphiregulin and neuregulin-1, or the intraocular injection of antibodies against the EGF receptor was associated with a reduction in axial elongation^6–8^. These observations led to the question whether the intraocular application of an antibody to the EGF receptor could clinically be helpful to prevent further axial elongation in adult patients with pathologic myopia^1–4^. The notion of EGF being involved in the process of axial elongation has been supported by a recent small case series study in which the intraocular concentration of EGF was higher in the eyes of highly myopic patients than in non-highly myopic eyes^9^.

In clinical oncology, EGF receptor antibodies, such as cetuximab, have systemically been used for two decades for the therapy of metastatic colorectal cancer and head and neck cancer^10,11^. In view of the long-standing experience gained with the intravenous application of EGF receptor antibodies with respect to their safety and side effects, there may be a potential to use EGF receptor antibodies applied intravitreally for the prevention of further axial elongation in adult highly myopic patients with myopic maculopathy and previous axial elongation. Since the intraocular administration of a previously intravenously applied drug may harbor unexpected risks and side effects, we conducted this study to examine the safety of intraocularly applied cetuximab, as an example of an EGF receptor antibody, in young and adult rabbits.

## Methods

The experimental study included a group of young rabbits (gray Soviet chinchilla) with an age of 8 weeks and a body weight of 1.6 kg, and a group of adult rabbits with an age of 18 weeks and a body weight of 2.4 kg. All rabbits were treated in accordance with the ARVO (Association for Research in Vision and Ophthalmology) Statement for the Use of Animals in Ophthalmic and Vision Research. The study was approved by the Ufa Eye Research Institute Biomedical Ethics Committee and is reported in accordance with the ARRIVE (Animal Research: Reporting of In Vivo Experiments) guidelines. The rabbits were obtained from a commercial vendor (Federal State Unitary Enterprise ‘‘Scientific and Production Association for Immunological Preparations ‘‘Microgen’’ of the Ministry of Health of the Russian Federation, Ufa, Bashkortostan, Russia). The animals were kept at a constant temperature (22 ± 1 °C) and in a light-controlled environment (lights on from 7 am to 7 pm) with ad libitum access to food and water.

The rabbits received three intravitreal injections of 0.5 mg cetuximab (Erbitux; Merck Healthcare KGaA, Darmstadt, Germany) in 0.10 mL into their right eyes in one-month intervals, while the left eyes received an intravitreal injection of 0.10 mL of Ringer's solution (Gematek OOO Company, Moscow, Russia). Directly after each injection, the intraocular (IOP) was measured in both eyes. All animals underwent regular ophthalmological examinations at baseline, the first day after the injection, and at four-week intervals. The examinations included inspection of the external eye, tonometry (Auto-2Ref/Keratometer HRK-7000A Huvitz Co, Ltd., Gyeonggi-do, Korea), and fundus photography (VISUCAM 500, Carl Zeiss Meditec AG, Jena Germany). We applied ocular biometry (Compact Touch Quantel Medical, Cournon d’Auvergne, France) to measure the axial length, the anterior chamber depth, and lens thickness. As described previously, the injections were performed in the upper right quadrant of the eyes at a distance of 3–4 mm from the limbus under general anesthesia^12^. Anesthesia was achieved by an intramuscular injection (biceps femoris) of Zoletil (15 mg/kg) (tiletamine mixed with zolazepam; Valdepharm Co., Val-de-Reuil, France) and xylazine (20 mg/kg) (Xyla; Interchemie Werken, De Adelaar B.V., A Waalre, The Netherlands) and additional by the topical application of anesthetic eye drops (0.4% oxybuprocain, Inocain; Promed Exports, New Delhi, India). All animals underwent both anesthesia methods simultaneously applied in the same manner.

Four weeks after the third injection, the eyes were enucleated under deep general anesthesia, and the rabbits were sacrificed by injecting air (5–10 mL) into the ear vein. We used a mixture of 4% formaldehyde and 1% glutaraldehyde to fixate the globes immediately after they had been enucleated. As described in detail previously, we measured the globe dimensions in the anterior–posterior direction, horizontal direction, and vertical direction^12^. For each eye, we removed a central part which was about 8 mm thick and included the pupil and the optic nerve head. It was dehydrated in alcohol, before we imbedded it in paraffin. We prepared slides with a thickness of 4–6 µm, and stained them with hematoxylin eosin. We measured the thickness of the outer nuclear layer, the inner nuclear layer, the combined inner nuclear layer, outer plexiform layer and outer nuclear layer, and the total retina at the posterior pole, at the midpoint between the posterior pole and equator, at the equator, and close to the ora serrata.

Using a statistical software package (SPSS for Windows, version 27.0, IBM-SPSS, Chicago, IL, USA), we assessed the mean values ± their standard deviations of the main outcome parameters, i.e., intraocular pressure, the histomorphometric parameters and ocular length. We calculated differences in these parameters between right eyes (study eyes) and the left eyes (control eyes) and assessed the statistical significance of these differences using the student t-test for paired samples or the Wilcoxon test. Since a multitude of statistical comparisons were performed, we corrected the results using Bonferroni's method with multiplying the P-value with the number of comparisons performed. All *P*-values were two-sided and were considered statistically significant when the values were less than 0.05.

## Results

The study included 10 adult rabbits with a mean age of 18 weeks (range: 17–19 weeks) and 8 young rabbits with a mean age of 8 weeks (range: 7–10 weeks).

In the young animal group, axial length increased significantly during the study period from 15.6 ± 0.4 mm at baseline to 16.3 ± 0.4 mm at study end in the right eyes (*P* = 0.01), and from 15.8 ± 0.6 mm to 16.5 ± 0.4 mm in the left eyes (*P* = 0.04). In the adult group, axial length did not change significantly during the study period (right eyes: from 16.4 ± 0.7 mm to 16.1 ± 0.3 mm; *P* = 0.20; left eyes: 16.3 ± 0.5 mm to 16.2 ± 0.6 mm; *P* = 0.79).

Neither in the young animal group nor in the adult rabbit group did the biometric measurements of axial length, anterior chamber depth and lens thickness and the IOP readings differ significantly (all *P* values > 0.05, Bonferroni corrected) between the right (study) eyes and the left (control) eyes (Table 1).

**Table 1:** Biometric measurements and intraocular pressure (IOP) readings (mean ± standard deviation) in the right (study) eyes (with repeated intravitreal injections of cetuximab) and the left (control) eyes with (repeated intravitreal injections of Ringer's solution) of young rabbits (Table 1a) and adult rabbits (Table 1b).

	Right eye	Left eye	*P* value
**a. Young rabbits**			
**Axial length (mm)**			
Baseline and first injection	15.61 ± 0.36	15.80 ± 0.59	0.51
Follow-up 4 weeks and second injection	15.95 ± 0.35	15.83 ± 0.35	0.56
Follow-up 8 weeks and third injection	15.88 ± 0.47	15.87 ± 0.40	0.94
Follow-up 12 weeks and study end	16.29 ± 0.41	16.51 ± 0.35	0.04
Change in axial length (mm)	0.69 ± 0.55	0.71 ± 0.78	0.95
**Anterior chamber depth (mm)**			
Baseline and first injection	1.84 ± 0.19	1.68 ± 0.14	0.14
Follow-up 4 weeks and second injection	1.98 ± 0.51	1.68 ± 0.12	0.17
Follow-up 8 weeks and third injection	2.00 ± 0.48	1.67 ± 0.13	0.12
Follow-up 12 weeks and study end	1.88 ± 0.14	2.10 ± 0.30	0.12
** Lens thickness (mm)**			
Baseline and first injection	2.40 ± 0.50	3.10 ± 1.17	0.19
Follow-up 4 weeks and second injection	2.34 ± 0.51	2.58 ± 0.79	0.49
Follow-up 8 weeks and third injection	2.40 ± 0.39	2.55 ± 0.78	0.64
Follow-up 12 weeks and study end	2.86 ± 0.52	3.19 ± 0.60	0.20
**Intraocular pressure (mmHg)**			
Baseline and first injection	11.4 ± 1.8	11.9 ± 0.6	0.45
At once after first injection	38.3 ± 15.5	31.4 ± 15.2	0.53
2.5 min after first injection	19.6 ± 9.7	18.5 ± 6.0	0.81
Follow-up 4 weeks and second injection	12.1 ± 0.7	11.9 ± 0.9	0.35
At once after second injection	51.8 ± 14.5	49.6 ± 12.9	0.78
2.5 min after second injection	51.4 ± 14.4	45.6 ± 13.8	0.41
Follow-up 8 weeks and third injection	10.4 ± 1.3	11.1 ± 1.1	0.27
At once after third injection	41.9 ± 20.5	47.9 ± 11.1	0.48
2.5 min after third injection	28.1 ± 14.7	39.5 ± 11.8	0.046
Follow-up 12 weeks and study end	10.3 ± 0.7	12.3 ± 1.4	0.02
**b. Adult rabbits**			
**Axial length (mm)**			
Baseline and first injection	16.38 ± 0.65	16.26 ± 0.50	0.69
Follow-up 4 weeks and second injection	16.26 ± 0.38	16.14 ± 0.59	0.46
Follow-up 8 weeks and third injection	16.44 ± 0.60	16.26 ± 0.47	0.55
Follow-up 12 weeks and study end	16.06 ± 0.28	16.24 ± 0.47	0.42
Change in axial length (mm)	− 0.31 ± 0.66	− 0.07 ± 0.75	0.53
**Anterior chamber depth (mm)**			
Baseline and first injection	1.87 ± 0.37	1.99 ± 0.42	0.12
Follow-up 4 weeks and second injection	1.74 ± 0.18	1.72 ± 0.12	0.85
Follow-up 8 weeks and third injection	1.80 ± 0.18	1.81 ± 0.16	0.86
Follow-up 12 weeks and study end	1.75 ± 0.06	1.76 ± 0.06	0.57
**Lens thickness (mm)**			
Baseline and first injection	2.39 ± 0.57	2.46 ± 0.67	0.79
Follow-up 4 weeks and second injection	2.18 ± 0.68	2.10 ± 0.50	0.81
Follow-up 8 weeks and third injection	2.02 ± 0.44	2.40 ± 1.02	0.31
Follow-up 12 weeks and study end	2.22 ± 0.45	2.31 ± 0.65	0.78
**Intraocular pressure (mmHg)**			
Baseline and first injection	12.2 ± 1.1	13.3 ± 2.0	0.06
At once after first injection	41.6 ± 18.4	40.7 ± 16.1	0.69
2.5 min after first injection	32.1 ± 15.4	29.3 ± 15.9	0.65
Follow-up 4 weeks and second injection	11.9 ± 1.1	12.3 ± 0.6	0.35
At once after second injection	43.1 ± 17.1	44.8 ± 18.0	0.84
2.5 min after second injection	40.6 ± 17.1	43.1 ± 16.1	0.72
Follow-up 8 weeks and third injection	11.1 ± 1.2	11.6 ± 1.0	0.24
At once after third injection	23.2 ± 17.3	45.1 ± 19.3	0.06
2.5 min after third injection	16.4 ± 11.3	42.2 ± 18.5	0.001
Follow-up 12 weeks and study end	11.0 ± 1.1	12.0 ± 1.0	0.11

None of the eyes showed during the study period an intraocular inflammation or peculiarities of the fundus, such as intraretinal inflammatory infiltrations. Comparing in a masked manner photographs of the anterior segment and of the fundus, taken at study end and at baseline, did not reveal any detected difference during the follow-up.

Histomorphometry revealed that the thickness of the outer nuclear layer, the inner nuclear layer, the combined outer nuclear layer, outer plexiform layer and inner nuclear layer, and the total retina, as measured at the posterior pole, the posterior pole/equator midpoint, the equator, and close to the ora serrata did not vary significantly between the study eyes and the control eyes (all *P* > 0.05, with Bonferroni correction) (Table 2).

**Table 2:** Histomorphmetrically measured thickness (mean ± standard deviation) of the outer nuclear layer, inner nuclear layer, the inner nuclear layer, outer plexiform layer and outer nuclear layer combined, and the total retinal thickness at the posterior pole, at the midpoint between the posterior pole and equator, at the equator, and close to the ora serrata.

	Right (study eyes)	Left (control) eyes	*P* value
**Outer nuclear layer thickness**			
Posterior pole	35.3 ± 7.3	30.7 ± 4.9	0.14
Midpoint posterior pole to equator, side A	27.8 ± 4.5	25.3 ± 5.0	0.38
Midpoint posterior pole to equator, side B	27.0 ± 5.7	26.6 ± 4.1	0.64
Equator, side A	25.5 ± 6.6	23.0 ± 6.4	0.39
Equator, side B	23.6 ± 7.2	24.4 ± 6.5	0.68
Close to ora serrata, side A	22.1 ± 5.3	19.3 ± 6.6	0.07
Close to ora serrata, side B	19.3 ± 10.4	19.5 ± 5.8	0.71
**Inner nuclear layer**			
Posterior pole	22.9 ± 8.9	14.0 ± 4.2	0.01
Midpoint posterior pole to equator, side A	15.0 ± 7.0	11.3 ± 2.0	0.20
Midpoint posterior pole to equator, side B	14.1 ± 4.5	12.8 ± 4.7	0.47
Equator, side A	12.8 ± 5.3	9.7 ± 3.9	0.17
Equator, side B	11.1 ± 2.9	13.1 ± 5.8	1.00
Close to ora serrata, side A	11.6 ± 5.2	11.7 ± 4.1	1.00
Close to ora serrata, side B	9.0 ± 3.9	10.5 ± 3.6	0.85
**Inner nuclear layer, outer plexiform layer and outer nuclear layer combined**			
Posterior pole	72.8 ± 25.0	55.3 ± 10.7	0.08
Midpoint posterior pole to equator, side A	53.6 ± 14.7	46.7 ± 9.3	0.21
Midpoint posterior pole to equator, side B	51.0 ± 11.5	47.6 ± 10.6	0.40
Equator, side A	49.5 ± 11.8	43.3 ± 10.2	0.14
Equator, side B	45.4 ± 11.8	45.8 ± 12.2	0.49
Close to ora serrata, side A	44.6 ± 14.1	38.0 ± 10.7	0.06
Close to ora serrata, side B	36.4 ± 15.5	35.6 ± 7.9	0.62
**Total retinal thickness**			
Posterior pole	137.3 ± 29.8	105.3 ± 17.7	0.04
Midpoint posterior pole to equator, side A	112.1 ± 29.4	92.0 ± 22.2	0.14
Midpoint posterior pole to equator, side B	105.0 ± 30.2	87.4 ± 15.2	0.14
Equator, side A	106.5 ± 36.1	82.3 ± 14.1	0.06
Equator, side B	94.7 ± 31.4	82.1 ± 19.6	0.20
Close to ora serrata, side A	93.8 ± 30.1	69.3 ± 19.3	0.01
Close to ora serrata, side B	76.7 ± 35.4	70.9 ± 14.7	0.58

## Discussion

This experimental study on young and adult rabbits did not show any inflammatory or otherwise destructive process associated with the repeated intravitreal application of cetuximab. The study group of eyes with the cetuximab injection as compared to the contralateral eyes with an intraocular injection of Ringer's solution did not differ significantly (all *P* values > 0.05, Bonferroni corrected) in any biometric parameter such as axial length or anterior chamber depth nor in IOP. During the study period, intraocular inflammatory changes were not detected. Histomorphometry did not reveal any significant difference (all *P* values > 0.05, Bonferroni corrected) between study eyes and control eyes in the thickness of the various retinal layers and the total retina at the posterior pole and other ocular regions. The findings suggest that the intraocular application of cetuximab was tolerated.

The results of this study cannot directly be compared with findings obtained in other investigations since the safety of intraocular cetuximab applications has not been explored previously. The findings obtained in this investigation indirectly concur with the observations made with the intravenous application of cetuximab in patients undergoing therapy of metastatic colorectal cancer or advanced squamous cell carcinoma of head and neck with EGFR expression^13,14^. While the most commonly observed side effects of the systemic therapy with cetuximab were cutaneous rash/desquamation, fatigue and nausea, side effects in the central nervous system including the eyes were not commonly detected^10,11,13,14^.

The dose of cetuximab used in our study was 0.5 mg Erbitux in 0.10 mL. Taking into account the dimensions of the adult rabbit eye with a diameter of approximately 16 mm and a calculated volume of approximately 2.1 cm^3^, the dose of 0.5 mg of cetuximab injected into the rabbit eyes corresponds to a dose of 3.0 mg cetuximab (Erbitux) (in 0.6 mL) injected into a human highly myopic eye with an axial length of about 29 mm and a calculated volume of about 12.8 cm^3^. The systemically applied dose of cetuximab for oncological reasons is 400 mg cetuximab/m^2^ body surface/week or 688 mg cetuximab for a normal adult. Considering that a highly myopic eye with a volume of about 13 cm^3^ makes out about 1/5000 of the body weight and volume, the dose of an intraocular application, comparable with the systemic application, would be 688 mg cetuximab/5000 or 0.14 mg cetuximab. The dose used in our study and adjusted for the volume of a human myopic eye was 3.0 mg cetuximab. This figure is about 20 times higher than the figure of 0.14 mg cetuximab as an intraocular equivalent of a systemic application of cetuximab. Despite the 20 times higher dose of cetuximab used in our experimental study, we did not detect intraocular side effects in the rabbit eyes.

The concentration of cetuximab as an EGF receptor antibody used in the rabbits of this study (500 µg cetuximab/2100 mm^3^ rabbit ocular volume or 0.24 ug cetuximab/mm^3^ rabbit ocular volume) was higher than the intraocular concentration of an EGF receptor antibody applied in a recent study, in which guinea pigs with a globe diameter of 8 mm and a globe volume of 268 mm^3^ received a dose of 20 µg of the EGF receptor antibody (20 µg EGF receptor antibody/268 mm^3^ or 0.07 µg EGF receptor antibody/mm^3^)^4^. As in the present study, the study on guinea pigs did not reveal any sign of intraocular toxicity of the intraocularly and repeatedly applied EGF receptor antibody. In the latter study, the EGF receptor antibody was specific for guinea pig EGF receptors. The present study thus adds the information to the current knowledge, that the intraocular and repeated application of cetuximab (Erbitux including its expedients) as another EGF receptor antibody is intraocularly tolerated in the doses applied in the study.

The infusion bottle of Erbitux contains, besides cetuximab, the expedients of NaCl, glycine, polysorbate 8, citrate acid monohydrate, and sodium hydroxide^15^. The observations made in our study suggest that these expedients injected in the dose as in our study are well tolerated intraocularly. It agrees also with observations made with the intraocular applications of drugs such as Avastin (bevacizumab; with the expedients of trehalose 2-H_2_O, sodium phosphate, and polysorbat-20), Lucentis (ranibizumab, with the expedients of trehalose-2-H_2_O, histidine hydrochloride-1-H_2_O, histidine, polysorbate 20, and water), Eylea (aflibercept, with the expedients of polysorbate 20 (E 432), sodium dihydrogen phosphate-1-H_2_O, di sodium hydrogen phosphate-7-H_2_O, sodium chloride, sucrose, and water), Beovu (brolucizumab, with the expedients of sodium citrate, sucrose, and polysorbate 80), Macugen (pegabtanib, with the expedients of sodium chloride, monobasic sodium phosphate monohydrate, dibasic sodium phosphate heptahydrate, sodium hydroxide, and hydrochloric acid), Miochol (sodium chloride 0.64%, with the expedients of potassium chloride 0.075%, calcium chloride dehydrate 0.048%, magnesium chloride hexahydrate 0.03%, sodium acetate trihydrate 0.39%, sodium citratedehydrate 0.17%, sodium hydroxide and/or hydrochloric acid, balanced Salt Solution (sodium chloride, potassium chloride, dibasic sodium phosphate, sodium bicarbonate, hydrochloric acid and/or sodium hydroxide, calcium chloride dihydrate, magnesium chloride hexahydrate, dextrose, glutathione disulfide), and VisionBlue (sodium mono-hydrogen orthophosphate, with the expedients of sodium di-hydrogen orthophosphate, sodium chloride). All these drugs contain similar expedients and are intraocularly well tolerated.

When the results of our study are discussed, its limitations have to be taken into account. First, as an experimental animal study, the findings obtained in the study cannot directly be transferred to human eyes. Second, cetuximab is an EGFR blocker focused on primates while it has a markedly lower affinity to the EGF receptor in other species. The effect of a blockade of the intraocular EGF receptor by intravitreally applied cetuximab was thus not fully explored in the present study. Correspondingly, the physiologic eye growth in the young rabbit group was not significantly influenced by the intravitreal cetuximab application. It is in contrast to studies with young guinea pigs in which the physiological eye growth and the lens-induced myopic enlargement of the globe were reduced when an EGF receptor antibody specific for guinea pig EGF receptors was intraocularly injected^4^. It was also in contrast to findings obtained in young nonhuman primates in which intravitreally amphiregulin antibody as another EGF antibody was associated with a reduction of the physiological eye growth (own data). Fourth, our study included ocular biometry, tonometry and morphological evaluations, while functional tests such as electroretinography were not performed. Such methods could have yielded additional information about the tolerability and safety of cetuximab intravitreally applied.

In conclusion, the repeated intravitreal application of cetuximab did not result in any detected intraocular toxic or destructive effects in young and adult rabbits. The results do not contradict the intravitreal use of cetuximab in patients.

## Data Availability

The datasets used and/or analyzed during the current study are available from the corresponding author on reasonable request.
